# Neighborhood deprivation in relation to lung cancer in individuals with type 2 diabetes—A nationwide cohort study (2005–2018)

**DOI:** 10.1371/journal.pone.0288959

**Published:** 2023-07-21

**Authors:** Xinjun Li, Filip Jansåker, Jan Sundquist, Casey Crump, Tsuyoshi Hamano, Kristina Sundquist

**Affiliations:** 1 Center for Primary Health Care Research, Department of Clinical Sciences Malmö, Lund University, Malmö, Sweden; 2 Department of Clinical Microbiology, Center of Diagnostic Investigations, Rigshospitalet, Copenhagen, Denmark; 3 Department of Family Medicine and Community Health, Department of Population Health Science and Policy, Icahn School of Medicine at Mount Sinai, New York, New York, United States of America; 4 Center for Community-Based Healthcare Research and Education (CoHRE), Organization for Research and Academic Information, Shimane University, Matsue, Shimane, Japan; 5 Department of Sports Sociology and Health Sciences, Kyoto Sangyo University, Kyoto, Japan; Aarhus University, DENMARK

## Abstract

**Background:**

Neighborhood deprivation has been found associated with both type 2 diabetes and lung cancer. The aim of this study was to examine the potential association between neighborhood deprivation and lung cancer incidence or mortality in individuals diagnosed with type 2 diabetes. The results may identify a new risk or prognostic factor for lung cancer in this important subgroup and help develop a more contextual approach to prevention that includes neighborhood environment.

**Methods and findings:**

The study population included adults (n = 613,650) aged ≥ 30 years with type 2 diabetes during 2005 to 2018 in Sweden. Cox regression was used to compute hazard ratios (HRs) and 95% confidence intervals (95% CIs) for incidence or mortality of lung cancer associated with neighborhood deprivation. All models were conducted in both men and women and adjusted for individual-level characteristics (e.g. age, smoking- and alcohol-related comorbidities, sociodemographic factors). The cumulative incidence and mortality for lung cancer were 1.08% (95% CI, 1.06 to 1.11) and 0.93% (0.90 to 0.95), respectively, in the study population during the study period. Neighborhood deprivation was associated with both incidence and mortality of lung cancer in patients with type 2 diabetes independently of the individual-level characteristics. In the fully adjusted models, comparing high- with low-deprivation neighborhoods, the HRs for lung cancer incidence were 1.21 (1.10 to 1.33) in men and 1.08 (0.95 to 1.21) in women. The corresponding HRs for lung cancer mortality were 1.04 (1.00 to 1.07) in men and 0.97 (0.94 to 1.00) in women. Competing risk analyses including cardiovascular mortality attenuated the results.

**Conclusion:**

In this large cohort of individuals with type 2 diabetes, we found higher lung cancer incidence and mortality in patients living in areas with high neighborhood deprivation, even after adjusting for individual-level characteristics. These findings may help develop a more contextual approach that includes the neighborhood environment when allocating resources for disease prevention and care in patients with type 2 diabetes. These findings could also help inform clinical care for patients with type 2 diabetes, particularly those living in deprived neighborhoods.

## Introduction

Type 2 diabetes has a high global health burden because of its increasing prevalence and many associated comorbidities [1]. Both type 2 diabetes and neighborhood deprivation have previously been associated with increased risk of lung cancer [2, 3]. However, no studies have examined whether neighborhood deprivation is related to risk of lung cancer in persons with type 2 diabetes. This is important as such knowledge could help inform strategies for cancer prevention and survival in the growing number of persons worldwide with type 2 diabetes.

A large body of research has explored the association between type 2 diabetes and cancer. Epidemiological studies have reported positive associations between type 2 diabetes and many type of cancers, including colorectal, breast, endometrial, and pancreatic malignancies [4]. However, the potential association between diabetes mellitus and lung cancer risk or mortality is somewhat inconclusive [2, 5, 6]. A meta-analysis by Yi *et al*. [2] suggested that diabetes mellitus is associated with increased incidence of lung cancer in women but not in men. In addition, other investigators have reported that diabetes mellitus is associated with improved survival among patients with lung cancer [7], which partly might be attributed to the antidiabetic drug metformin [8].

Neighborhood deprivation has been associated with both diabetes mellitus and lung cancer. For example, studies have shown that the prevalence of type 2 diabetes is higher in deprived neighborhoods compared to affluent neighborhoods, even after adjusting for individual-level characteristics [9–11]. Furthermore, neighborhood deprivation has been associated with both lung cancer incidence and mortality [3, 12], as well as higher prevalence of major risk factors such as smoking [13]. However, the association between neighborhood deprivation and lung cancer in individuals with type 2 diabetes remains unknown. A better understanding of this association is needed to further identify individuals at high risk for lung cancer incidence or mortality and may reveal new strategies for cancer prevention and care in individuals with type 2 diabetes, one of the most common and serious chronic disorders. Therefore, we sought to assess the association between neighborhood deprivation and lung cancer in a nationwide follow-up study of patients with type 2 diabetes.

The aims of this study were to investigate (1) whether there is a difference in lung cancer incidence or mortality among women or men with type 2 diabetes living in deprived neighborhoods compared to those in affluent neighborhoods, and (2) whether neighborhood deprivation is an independent risk factor for lung cancer incidence or mortality in persons with type 2 diabetes after adjusting for individual-level potential confounders (age, marital status, family income, education, immigration status, urban/rural status, mobility, smoking, and comorbidities) [14, 15].

## Methods

### Study design and setting

A nationwide open cohort study was conducted of men and women ≥ 30 years of age with type 2 diabetes in Sweden, a country with universal healthcare provided to all residents [16]. The study period was from January 1, 2005 to December 31, 2018. Baseline was defined when an individual was diagnosed with type 2 diabetes. The STROBE statement-checklist [17] for cohort studies was considered when conducting the study and writing the manuscript. The research was conducted at Lund University, Sweden.

### Data sources

Data used in this study were retrieved from national registers of high quality and completeness [18–22]. These are comprehensive resources of data collected by the Swedish authorities, which contain individual-level information on all people in Sweden, including age, sex, socioeconomic status, occupation, geographical region of residence, hospital diagnoses and dates of hospital admissions in Sweden, date of emigration, and date and cause of death. The data sources were linked using the national 10-digit civic registration number, which is assigned to each individual in Sweden upon birth or immigration to the country. Our research group only had access to the pseudonymized version of this number (to ensure the integrity of all individuals). The following registers were used: Swedish Cancer Register [19] (1958–2018); National Patient Register (NPR) [20] (In-Patient data 1964–2018 and Out-Patient data 2001–2015); Cause of Death Register [22] (1961–2018); Total Population Register [21] (1968–2018); and Swedish Prescribed Drug Register [18] (2005–2018). Diagnoses and medical drugs were reported according to the different versions of the International Classification of Diseases (ICD) and the Anatomic Therapeutic Chemical (ATC) codes (S1 Table). The clinical data linked to national sociodemographic data were highly complete (less than one percent were missing). Missing values for sociodemographic data were also low for the study population: 0.0% values were missing for country of origin; 3.5% for educational attainment; 3.8% for family income; 3.5% for region of residency; and 1.4% for the neighborhood deprivation index.

### Ascertainment of study population

Individuals 30 years or older with either a diagnosis of type 2 diabetes or a redeemed prescription of an antidiabetic drug (whichever came first) during the study period were included in the study population. Each individual could be included only once. The Swedish Prescribed Drug Register [18] was used to identify all individuals aged 30 years and older with medically treated diabetes mellitus. This register includes all medical prescriptions that were retrieved at any pharmacy in Sweden between January 1, 2005, and December 31, 2018. All individuals that had been prescribed and redeemed insulin or oral antidiabetic agents (ATC-codes A10) during the entire time period between January 1, 2005, and December 31, 2018, were included in the study population. In addition, we used the main diagnoses for type 2 diabetes recorded in the NPR. In the present study, the first-time registration in the NPR of type 2 diabetes (primary or secondary diagnosis) was defined as a case according to ICD-10 codes E11 during the study period. In total, we identified 760,597 individuals 30 years and older that had redeemed an antidiabetic drug or received a type 2 diabetes diagnosis during the study period, whichever came first. We excluded 470 individuals previously diagnosed with lung cancer (catchment period: 2000–2004) and 1205 individuals diagnosed with lung cancer before type 2 diabetes during the study period. We also excluded all individuals diagnosed with type 1 diabetes (ICD-10 E10: 139,708 individuals) and gestational diabetes (ICD-10 O24: 5564 individuals) during the study period. A total of 613,650 individuals were judged to have type 2 diabetes and defined the study population.

### Ascertainment of outcome variables

The Swedish Cancer Register (ICD-7 162, 163) and Cause of Death Register (ICD-10 C33, C34) were used to identify the outcome variable of lung cancer incidence and lung cancer mortality (cause-specific for lung cancer) during the study period (2005–2018). Lung cancer incidence was identified in the Swedish Cancer Registry (established in 1958). In this register all cancer diagnoses for coding site have been translated to comply with ICD-7 [19]. Lung cancer mortality was identified in the Swedish Cause of Death Register (established in 1961) as either primary- or secondary cause of death [22].

### Ascertainment of neighborhood deprivation (main exposure)

#### Neighborhood-level variable

The home addresses of all Swedish adults have been geocoded to small geographic units that have boundaries defined by similar types of buildings. These neighborhood areas are called small area market statistics (SAMS) and have an average of 1000 to 2000 residents and were used as proxies for neighborhoods, as has been done in previous research [23].

*Neighborhood Deprivation Index* is a summary measure that has previously been used to characterize neighborhood-level deprivation [3, 23]. For this, we identified deprivation indicators used by past studies to characterize neighborhood environments and then used a principal components analysis to select deprivation indicators in the Swedish national registers [24]. The following four variables were selected: low educational status (<10 years of formal education); low income (income from all sources, including that from interest and dividends, defined as less than 50% of individual median income) [25]; unemployment (not employed, excluding full-time students, those completing compulsory military service, and early retirees); and social welfare assistance. Each of the four variables loaded on the first principal component with similar loadings (+.47 to +.53) and explained 52% of the variation between these variables. A z score was calculated for each SAMS neighborhood [23]. The z scores, weighted by the coefficients for the eigenvectors, were then summed to create the index [26]. Higher scores reflect more deprived neighborhoods. The index was categorized into three groups: more than one standard deviation (SD) below the mean (low deprivation), within one SD of the mean (moderate deprivation), and more than one SD above the mean (high deprivation). The participants living in low deprivation neighborhoods (i.e., most affluent neighborhoods) were defined as the reference group. Moreover, only 0.6% of the patients with type 2 diabetes were excluded because of missing SAMS codes.

### Ascertainment of individual level factors (covariates)

*Comorbidities* were identified during the study period from the National Patient Register: chronic obstructive pulmonary disease (COPD) (J40–J47), alcoholism and alcohol-related liver disorders (F10 and K70), and tobacco abuse (F17, T65.2, Z71.6, Z72.0). All individual-level sociodemographic variables were assessed from the Swedish Total Population Register at the start of follow-up (at baseline) for each individual. *Age* was categorized into 30–49, 50–59, 60–59, 70–79, and ≥ 80 years of age. *Country of origin* was divided into two groups: born in Sweden and born outside of Sweden. *Educational level* was divided into three groups based on: completion of compulsory school or less (≤ 9 years), practical high school or some theoretical high school (10–12 years), or theoretical high school and/or college (> 12 years). *Employment status* was divided into employed and unemployed. *Family income* was based on the annual family income divided by the number of people in the family, i.e., individual family income per capita. This variable also took into consideration the ages of people in the family and used a weighted system in which children were given lower weights than adolescents and adults. *Mobility* was based on the length of time lived in a neighborhood, categorized as having lived in the neighborhood < 5 years or ≥ 5 years. *Sex* was categorized into men and women. *Marital status* was divided into two groups: married/cohabitating, and never married, widowed, or divorced. *Urban/rural status* was divided into three groups: large cities (Stockholm, Göteborg, Malmö), middle-sized towns, and small towns/rural areas. The chosen covariates were included because they can act as potential confounders based on previous studies [3, 13–15, 23].

### Statistical analysis

Descriptive characteristics and numbers/rates of first events of lung cancer incidence and mortality were calculated for the total study population by level of neighborhood deprivation, and for each of the covariates. Person-years were computed from the start of follow-up (baseline), i.e., from the first diagnosis of type 2 diabetes or the first redeemed prescription of an antidiabetic drug, until each individual’s first diagnosis of lung cancer/mortality of lung cancer, death, emigration, or the end of the study on December 31, 2018. Age was included as a linear effect in the adjustments. Cox proportional hazards models, stratified by sex, were used to estimate hazard ratios (HR) and 95% CIs for lung cancer incidence or mortality associated with neighborhood deprivation, adjusting for individual characteristics and comorbidities. The first model in the analysis was a univariate Cox regression performed for each variable. Secondly, multivariate Cox regression models, including the main predictor variable and covariates, were performed in a stepwise fashion. A full model without stratification by sex was also conducted. We also adjusted for age divided into categories, but this made no difference in the risk estimates. Interaction tests were performed to examine whether the association between neighborhood deprivation and incidence or mortality of lung cancer among patients with type 2 diabetes varied significantly by any of the individual characteristics, but no meaningful interactions were found. All statistical analyses were performed using SAS 9.4 (SAS Institute Inc.; Cary, NC, USA).

As a sensitivity analysis we considered the possible modifying effect of metformin, as prior evidence has suggested that metformin is associated with reduced risk of lung cancer incidence and mortality [8]. A sensitivity analysis was therefore performed to examine the association between neighborhood deprivation and lung cancer incidence or mortality in patients with type 2 diabetes after stratifying by whether or not they were treated with metformin during the study period. Data on medical treatments (ATC-codes) were collected from the Swedish Prescription Register with follow-up from 2005 to 2018 (S1 Table). Competing risk analyses for cardiovascular mortality (measured as ICD-10 codes I00-I9 in the Cause of Death Register) on the main analyses of incidence and mortality of lung cancer were also conducted.

### Ethical considerations

The present study was a non-intervention nationwide register study based on pseudonymized secondary data obtained from the Swedish authorities and was approved by the Ethical Review Board in Lund (Sweden). All methods were performed in accordance with the relevant guidelines and regulations.

## Results

A total of 613,650 individuals with type 2 diabetes were included (56.1% men). The median age at baseline was 66 years, the interquartile age range was 57 to 75 years, and the entire age range was 30 to 106 years (data not shown).

Table 1 shows the number of lung cancer events and mortality, and the cumulative incidence and mortality for lung cancer by neighborhood deprivation level in patients with type 2 diabetes During the follow-up period (mean follow-up, six years), there were 6654 and 5687 events of lung cancer incidence and mortality among patients with type 2 diabetes, respectively. S2 Table includes the characteristics of the study population and number of lung cancer events and mortality by all the covariates.

**Table 1:** Distribution of population, number of lung cancer events and mortality from lung cancer in men and women with type 2 diabetes, 2005–2018.

		Neighborhood deprivation level		
	Total	Low	Moderate	High
**Total population**				
No.	613,650	121,209	346,241	146,200
%		19.8	56.4	23.8
**Events of lung cancer**				
No.	6654	1249	3635	1770
%		18.8	54.6	26.6
**Events of lung cancer mortality**				
No.	5687	1033	3082	1572
%		18.2	54.2	27.6
**Cumulative incidence of lung cancer, % (95% CI)**	1.08 (1.06 to 1.11)	1.03 (0.97 to 1.09)	1.05 (1.02 to 1.08)	1.21 (1.16 to 1.26)
**Cumulative mortality from lung cancer, % (95% CI)**	0.93 (0.90 to 0.95)	0.85 (0.79 to 0.91)	0.89 (0.85 to 0.93)	1.08 (1.03 to 1.12)

CI: Confidence interval.

S3 Table shows an apparent gradient of higher cumulative incidence and mortality for lung cancer by increasing neighborhood deprivation in the total study population (visualized in S1 Fig); the same pattern also appeared in most subgroups. The cumulative probability of not dying from lung cancer across the follow-up period was also lower in patients living in high-deprivation neighborhoods (S2 Fig).

Table 2 demonstrates the hazard ratios (HRs) for lung cancer incidence in men and women with type 2 diabetes. The results show that living in high-deprivation neighborhoods is associated with significantly higher HRs for lung cancer incidence in men compared with their counterparts living in low-deprivation neighborhoods. The risk of lung cancer incidence was also elevated in women with type 2 diabetes living in high-deprivation neighborhoods but non-significant when controlling for all the individual-level factors. Living in moderate-deprivation neighborhoods was associated with significantly lower HRs in both men and women. These results are also visualized in S3 Fig.

**Table 2:** Hazard ratios (HR) and 95% confidence intervals (CI) for incidence of lung cancer in men and women with type 2 diabetes; results of Cox regression models.

	Men									Women								
	Model 1			Model 2			Model 3			Model 1			Model 2			Model 3		
	HR	95% CI		HR	95% CI		HR	95% CI		HR	95% CI		HR	95% CI		HR	95% CI	
**Neighborhood deprivation (ref. Low)**																		
Moderate	0.96	0.89	1.04	0.90	0.82	0.97	0.88	0.81	0.96	0.86	0.78	0.95	0.82	0.74	0.91	0.81	0.73	0.90
High	1.55	1.41	1.70	1.26	1.14	1.39	1.21	1.10	1.33	1.15	1.03	1.30	1.13	1.00	1.28	1.08	0.95	1.21
**Age (ref. 30–49 years)**																		
50–59	4.98	3.83	6.48	5.61	4.31	7.30	5.38	4.14	7.00	5.87	4.29	8.05	6.02	4.39	8.26	5.74	4.18	7.88
60–69	11.38	8.83	14.66	13.23	10.25	17.08	12.36	9.57	15.95	10.76	7.93	14.60	10.89	8.02	14.80	10.26	7.55	13.94
70–79	15.84	12.28	20.43	17.38	13.45	22.48	16.07	12.42	20.80	10.11	7.44	13.73	9.79	7.19	13.33	9.57	7.03	13.04
≥ 80	11.92	9.11	15.60	12.58	9.58	16.51	12.01	9.15	15.77	4.57	3.30	6.33	4.09	2.94	5.69	4.34	3.12	6.04
**Education attainment (ref.> 12 years)**																		
≤ 9 years				1.76	1.59	1.94	1.69	1.53	1.86				1.60	1.41	1.82	1.54	1.36	1.75
10–12 years				1.49	1.35	1.65	1.44	1.31	1.59				1.44	1.26	1.63	1.38	1.21	1.57
**Family income (ref. Highest quartiles)**																		
Low income				1.63	1.48	1.79	1.57	1.43	1.73				1.07	0.93	1.23	1.04	0.91	1.19
Middle-low income				1.23	1.12	1.35	1.18	1.07	1.29				1.08	0.94	1.23	1.02	0.89	1.17
Middle-high income				1.17	1.07	1.27	1.13	1.03	1.23				1.14	0.99	1.30	1.09	0.95	1.25
**Region of residence (ref. Large cities)**																		
Southern Sweden				0.87	0.81	0.94	0.87	0.81	0.94				0.86	0.78	0.94	0.87	0.80	0.96
Northern Sweden				1.10	1.01	1.19	1.14	1.05	1.24				1.07	0.97	1.19	1.13	1.02	1.25
**Marital status (ref. Married/cohabiting)**				0.92	0.86	0.98	0.90	0.84	0.96				1.09	1.00	1.19	1.02	0.93	1.11
**Country of origin (ref. Sweden)**				1.25	1.15	1.36	1.24	1.14	1.35				0.76	0.68	0.85	0.78	0.70	0.87
**Mobility (ref. Not moved)**				1.44	1.33	1.54	1.40	1.31	1.51				1.51	1.38	1.65	1.46	1.33	1.60
**Comorbidities**																		
Hospitalization of COPD (ref. Non)							2.64	2.45	2.85							3.22	2.96	3.51
Hospitalization of alcoholism and related liver disorders (ref. Non)							1.10	0.96	1.26							1.33	1.06	1.66
Hospitalization of tobacco abuse (ref. Non)							3.19	2.75	3.69							2.63	2.18	3.17

Model 1: Adjusted for age; Model 2: Adjusted for individual sociodemographic characteristics; Model 3: Full model (incl. comorbidities). HR: Hazard ratio; CI: Confidence interval; COPD: Chronic obstructive pulmonary disease.

**Fig 1:**
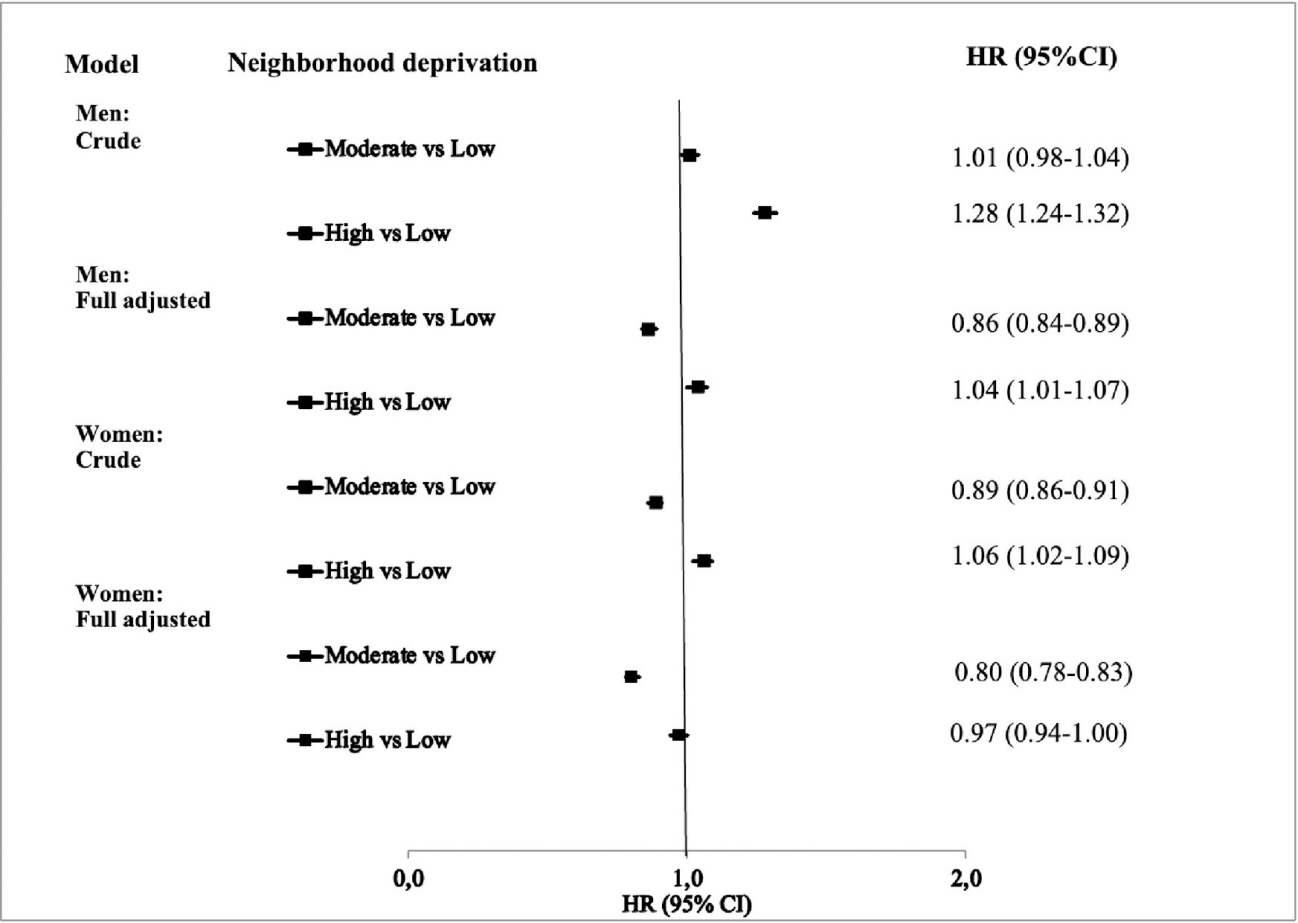
Hazards ratios (HR) and 95% confidence intervals (CI) for lung cancer mortality in men and women with type 2 diabetes. HR: Hazard ratio; CI: Confidence interval.

Fig 1 shows the HRs for lung cancer mortality in men and women with type 2 diabetes. After adjustment for individual-level factors, the HR for lung cancer mortality was significantly elevated for men with type 2 diabetes living in high-deprivation neighborhoods compared to low-deprivation neighborhoods. For women, the age-adjusted HR for lung cancer mortality was slightly elevated but the association disappeared after adjusting for the other individual-level factors. The risk was lower in moderate-deprivation neighborhoods in both men and women. The HRs are also shown in S4 Table (men) and S5 Table (women), which include the HRs associated with other individual-level factors as well.

In both men and women, some of the individual-level variables were associated with significantly increased lung cancer incidence or mortality in the full models (Table 2, S4 and S5 Tables). For example, lung cancer incidence and mortality were higher for men and women with low education, men born outside of Sweden, those who had moved, and those with comorbidities (especially COPD and tobacco abuse).

Table 3 includes the HRs for incidence and mortality of lung cancer in fully adjusted models that included both men and women. The HRs were 1.41 (95% CI 1.35–1.50) for lung cancer incidence and 1.50 (95% CI 1.41–1.58) for lung cancer mortality in male compared to female patients with type 2 diabetes. Moreover, significant associations between neighborhood deprivation and lung cancer incidence (HR 1.16; 95% CI 1.08–1.25) and mortality (HR 1.22; 95% CI 1.12–1.32) were observed in these models that were adjusted for sex.

**Table 3:** Hazard ratios (HR) and 95% confidence intervals (CI) for incidence and mortality of lung cancer; results of Cox regression models.

	Incidence lung cancer			Mortality lung cancer		
	HR	95% CI		HR	95% CI	
**Neighborhood deprivation (ref. Low)**						
Moderate	0.85	0.80	0.91	0.82	0.77	0.89
High	1.16	1.08	1.25	1.22	1.12	1.32
**Age (ref. 30–49 years)**						
50–59	5.43	4.44	6.65	5.85	4.62	7.42
60–69	11.28	9.27	13.73	12.69	10.08	15.98
70–79	12.81	10.52	15.61	16.33	12.96	20.57
≥ 80	7.70	6.24	9.50	14.16	11.15	17.99
**Gender to males (ref. Females)**	1.42	1.35	1.50	1.50	1.41	1.58
**Family income (ref. Highest quartiles)**						
Low income	1.35	1.25	1.46	1.72	1.58	1.88
Middle-low income	1.20	1.11	1.30	1.44	1.32	1.57
Middle-high income	1.18	1.10	1.27	1.31	1.21	1.43
**Education attainment (ref. ≥ 12 years)**						
≤ 9 years	1.59	1.47	1.72	1.78	1.63	1.95
10–11 years	1.41	1.30	1.52	1.47	1.35	1.61
**Country of origin (ref. Sweden)**	1.05	0.98	1.12	1.01	0.94	1.09
**Marital status (ref. Married/cohabiting)**	0.92	0.87	0.97	0.92	0.87	0.98
**Region of residence (ref. Large cities)**						
Southern Sweden	0.87	0.82	0.92	0.92	0.86	0.98
Northern Sweden	1.14	1.07	1.22	1.35	1.26	1.44
**Mobility (ref. Not moved)**	1.44	1.36	1.53	1.65	1.55	1.75
**Comorbidities**						
Hospitalization of COPD (ref. Non)	2.93	2.77	3.10	2.67	2.51	2.84
Hospitalization of alcoholism and related liver disorders (ref. Non)	1.12	1.00	1.26	1.25	1.10	1.42
Hospitalization of tobacco abuse (ref. Non)	3.03	2.70	3.40	2.50	2.18	2.88

Fully adjusted for all covariates. HR: Hazard ratio; CI: Confidence interval; COPD: Chronic obstructive pulmonary disease.

Additional analyses were conducted on the incidence and mortality of lung cancer in patients with type 2 diabetes treated with and without metformin. In S6 Table, comparing high- vs. low-deprivation neighborhoods, the HRs for lung cancer incidence were similar in patients treated with metformin (HR 1.18; 95% CI 1.07–1.30) and those who were not (HR 1.13; 95% CI 1.01–1.28). The corresponding HRs for lung cancer mortality in patients treated with metformin (HR 1.27; 95% CI 1.14–1.42) were also similar to those without metformin (HR 1.16; 95% CI 1.03–1.31). In S7 Table, comparing the patients with and without metformin treatment, the results show that metformin treatment was associated with a reduced risk of lung cancer incidence (HR 0.65; 95% CI 0.61–0.68) and lung cancer mortality (HR 0.56; 95% CI 0.53–0.60). This association was significant at all levels of neighborhood deprivation to a varying degree and most pronounced in the high-deprivation neighborhoods. The associations between neighborhood deprivation and lung cancer incidence and mortality were substantially weakened when taking the competing risks due to cardiovascular mortality into account (S8 Table).

## Discussion

In this nationwide cohort study of patients with type 2 diabetes, we found that lung cancer incidence and mortality were higher among those living in high-deprivation neighborhoods compared to those in low-deprivation neighborhoods. This difference was attenuated but remained significant after adjustment for the individual-level sociodemographic variables and residential mobility.

A previous epidemiological study showed disparities in overall cancer mortality in individuals based on their diabetes status and socioeconomic status (SES) [27]. Living in socioeconomically deprived neighborhoods has been associated with an increased risk of several morbidities, including https://www.sciencedirect.com/science/article/pii/S0167527315304666?via%3Dihub-bb0075lung cancer and diabetes mellitus [2, 3, 11, 28]. More specifically, we have previously shown that neighborhood deprivation is associated with lung cancer incidence and mortality [3]. Diabetes mellitus also seems to be associated with lung cancer but to a varying degree among men and women [2, 5, 6]. In this present study, we found that lung cancer incidence and mortality in patients with type 2 diabetes were significantly associated with neighborhood deprivation in both sexes (but most pronounced in men), even after adjusting for individual sociodemographic factors and smoking- or alcohol-related comorbidities.

The causal pathways between neighborhood deprivation and cancer are not fully understood. However, several possible mechanisms could explain our findings. Differences in lifestyle attitudes and beliefs across SES levels in patients with diabetes mellitus may be important contributors [29–32]. For instance, a study from the United Kingdom found that smoking was more common among patients with diabetes mellitus living in deprived neighborhoods than among those living in more affluent neighborhoods [30]. Similar results were found in another neighborhood study of mortality risk factors among patients with diabetes mellitus [32]. Another study showed that lung cancer risk factors such as smoking were more common in people living in deprived compared to affluent neighborhoods in Sweden [29]. In addition, sociocultural norms regarding diet, smoking and physical activity could also vary in different neighborhoods and affect the health of residents and their consequent risk for disease. Moreover, limited opportunities to participate in cancer prevention programs, including general health workups, and the resultant failure in the early detection of cancer may explain the higher cancer mortality rate in groups with low socioeconomic status. For example, a study conducted in Denmark found that living in more affluent neighborhoods was associated with a higher probability of participating in the health check-up phase of a population-based lifestyle intervention [33]. Therefore, it is possible that these risk factors may have contributed to the increase in cancer mortality in people with diabetes mellitus residing in high-deprivation neighborhoods.

Another possible explanation is that neighborhood deprivation affected the treatment of diabetes mellitus. A German study showed that social inequalities were associated with different antidiabetic drug treatments [34], which could be related to lung cancer [8]. However, the evidence regarding the association between metformin use and cancer risks in patients with type 2 diabetes is conflicting. Data regarding effects of diabetic therapy on lung cancer outcomes are sparse and retrospective. Some studies have found that metformin may improve chemotherapy outcomes for patients with non-small cell lung carcinoma compared with other therapies (e.g., insulin, sulfonylureas) [35, 36]. Other studies have suggested that metformin use has no impact or even might decrease survival for patients with lung cancer [37, 38]. Although Sweden has a universal health care system, it may be possible that there are neighborhood differences in the quality of healthcare, including access to medical treatment for type 2 diabetes that potentially may affect incidence/mortality of lung cancer in patients with type 2 diabetes as well. Neighborhood variations in the use of antidiabetic drugs [39] could also potentially explain our finding of a risk-reducing effect of metformin treatment that seemed to be more pronounced in patients living in high-deprivation neighborhoods. However, future studies need to explore the mechanisms before any firm conclusions can be made.

Our study has several limitations, including the lack of data on certain risk factors for lung cancer, such as air pollution, passive smoking, and (most importantly) individual smoking status. However, we addressed this partly in our analyses by adjusting for tobacco abuse and hospitalization for chronic lower respiratory disease as proxies for smoking history; the findings remained significant but were slightly attenuated after these adjustments. Nevertheless, as smoking status was not directly assessed in our study and tobacco smoking is higher in deprived neighborhoods [29], tobacco smoking could be an important residual confounder. Since controlling for smoking status in a robust manner remains difficult in large-scale epidemiological studies, future research exploring this potential mechanism might need to focus on smaller samples with more detailed smoking data. Moreover, we lacked data on body mass index or the severity of type 2 diabetes for individual patients. In the analysis stratified by metformin use, we identified metformin use at any point during the study period. This might have resulted in a time gap between the diagnosis of type 2 diabetes and the first redeemed metformin prescription, which is a potential source of bias. However, any such bias should be minimal as most patients with diabetes receive a prescription of metformin soon after their diagnosis. It is also important to note that certain individuals changed their place of residence and neighborhood SES during the follow-up. For example, many individuals were elderly, and it could be expected that some of these individuals would change residence after retiring or becoming widowed. However, we adjusted our analyses for residential mobility, and neighborhood deprivation remained associated with significantly higher lung cancer incidence and mortality in this cohort of patients with diabetes mellitus. Lastly, although we also report estimates for lung cancer incidence and mortality associated with covariates, such estimates should be interpreted with caution because they may have different confounders than the main exposure [40].

Nonetheless, the present study also has a number of important strengths. The nationwide cohort included practically all patients (30 years and older) with type 2 diabetes in Sweden during the study period, which may enhance generalizability of our findings. The personal identification number (pseudonymized to ensure integrity for all individuals) that is assigned to each individual in Sweden enabled nearly complete follow-up of all patients. As the outcome data were based on hospital diagnoses rather than self-reported data, recall bias was avoided. An additional key strength was the access to small geographical units, SAMS (on the order of 1000–2000 persons) that consisted of relatively homogenous types of buildings. This further strengthens the generalizability of our results as small neighborhood units have previously been shown to correspond well with how the residents within these units define their neighborhoods [41]. The findings of previous studies, together with those of the present study, highlight the need for improving health in low resource settings, which is underway in Europe [42].

In conclusion, high neighborhood deprivation may be an independent risk factor for lung cancer incidence and mortality in women and men with type 2 diabetes. These findings may be useful in clinical care and cancer prevention in patients with type 2 diabetes, and particularly in those living in the most deprived neighborhoods. Understanding the pathways linking neighborhood factors with cancer outcomes is vital for developing a more contextual approach that includes the sociocultural and residential environment for allocation of healthcare resources to lung cancer prevention. Future research could also focus on the specific pathways by which neighborhood environments influence lung cancer incidence and mortality and how to reduce inequalities by neighborhood deprivation in patients with type 2 diabetes.

## Supporting information

S1 Fig Cumulative incidence (%) and mortality (%) for lung cancer among patients with type 2 diabetes by neighborhood deprivation index (2005–2018). (PDF)

S2 Fig The Kaplan–Meier curves for the probability of survival without lung cancer mortality for different levels of neighborhood deprivation in patients with type 2 diabetes. (PDF)

S3 Fig Hazard ratios (HR) and 95% confidence intervals (CI) for incidence for lung cancer in men and women with type 2 diabetes. (PDF)

S1 Table ICD-codes of diagnoses and ATC-codes for treatments. (DOC)

S2 Table Study population characteristics, number of lung cancer events and mortality for lung cancer (2005–2018). (DOC)

S3 Table **a.** Cumulative incidence (%) for lung cancer in patients with type 2 diabetes by levels of neighborhood deprivation (2005–2018). **b.** Cumulative mortality (%) for lung cancer in patients with type 2 diabetes by levels of neighborhood deprivation (2005–2018).
(DOC)

S4 Table Hazard ratios (HR) and 95% confidence intervals (CI) for mortality of lung cancer in men with diabetes mellitus; results of Cox regression models. (DOC)

S5 Table Hazard ratios (HR) and 95% confidence intervals (CI) for mortality of lung cancer in women with diabetes mellitus; results of Cox regression models. (DOC)

S6 Table Hazard ratios (HR) and 95% confidence intervals (CI) for incidence and mortality for lung cancer in patients diagnosed with type 2 diabetes with or without metformin treatment; results of Cox regression models. (DOC)

S7 Table Hazard ratios (HR) and 95% confidence intervals (CI) for lung cancer incidence and mortality of individuals with type 2 diabetes with metformin treatment compared to individuals with type 2 diabetes without metformin treatment. (DOC)

S8 Table Hazard ratios (HR) and 95% confidence intervals (CI) for incidence and mortality of lung cancer with competing risk for cardiovascular mortality; results of Cox regression models. (DOC)

## Abbreviations

ATC: Anatomic Therapeutic Chemical (codes)
CI: Confidence Interval
COPD: Chronic Obstructive Pulmonary Disease
HR: Hazard Ratio
ICD: International Classification of Diseases
SAMS: Small Area Market Statistics
SES: Socioeconomic Status
