# Supplementary material for: Neighborhood deprivation in relation to lung cancer in individuals with type 2 diabetes—A nationwide cohort study (2005–2018)
Source: PLoS One. 2023 Jul 21;18(7):e0288959. doi: 10.1371/journal.pone.0288959 (PMC10361504; doi:10.1371/journal.pone.0288959)
Supplement: S1 Fig — (PDF) [file pone.0288959.s001.pdf]

S1 Fig. Cumulative incidence and mortality for lung cancer among patients with type 2 diabetes by neighborhood deprivation index (2005-2018).

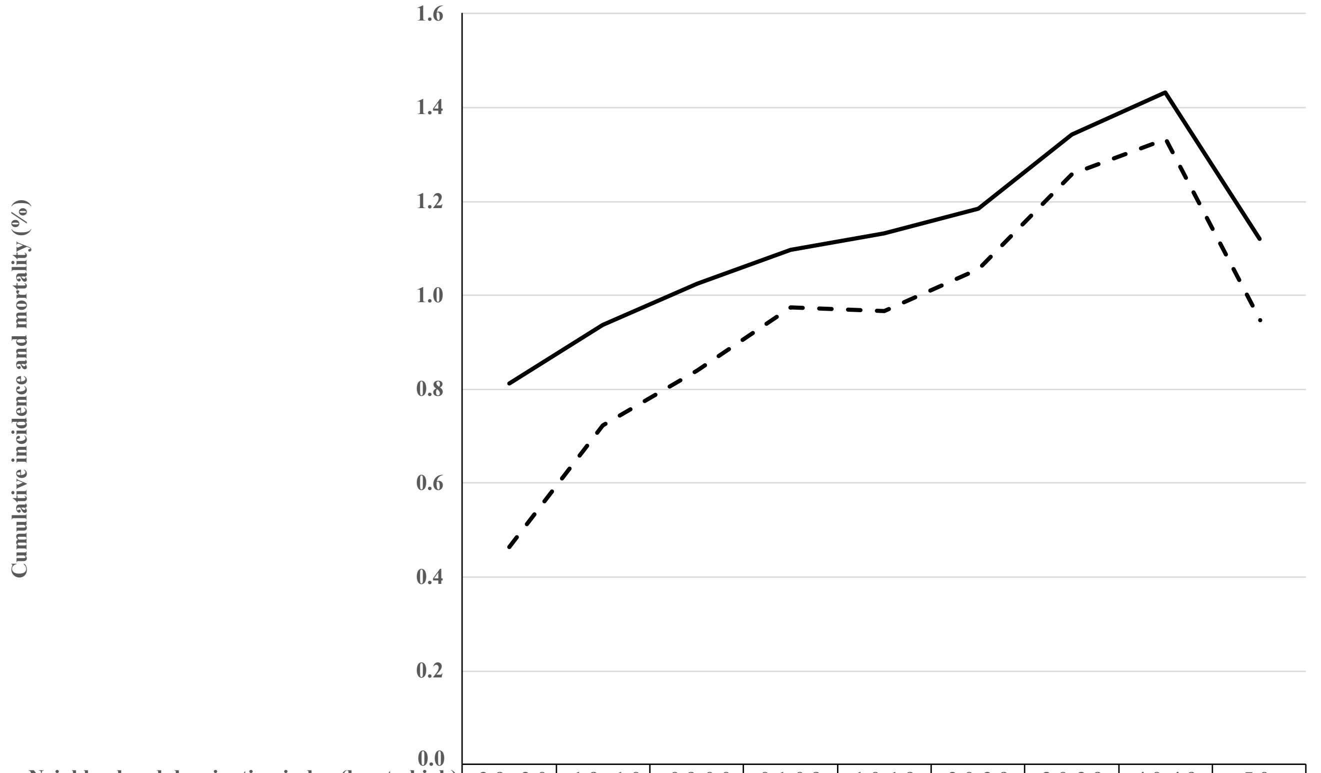

Neighborhood deprivation index (low to high)	-2.9--2.0	-1.9--1.0	-0.9-0.0	0.1-0.9	1.0-1.9	2.0-2.9	3.0-3.9	4.0-4.9	5.0-
Cumulative incidence of lung cancer (%)	0.81	0.94	1.03	1.10	1.13	1.19	1.34	1.43	1.12
Mortality of lung cancer (%)	0.46	0.72	0.84	0.97	0.97	1.06	1.26	1.33	0.95
