# Supplementary material for: Neighborhood deprivation in relation to lung cancer in individuals with type 2 diabetes—A nationwide cohort study (2005–2018)
Source: PLoS One. 2023 Jul 21;18(7):e0288959. doi: 10.1371/journal.pone.0288959 (PMC10361504; doi:10.1371/journal.pone.0288959)

S2 Fig. The Kaplan–Meier curves for the probability of survival without lung cancer mortality for different levels of neighborhood deprivation in patients with type 2 diabetes.

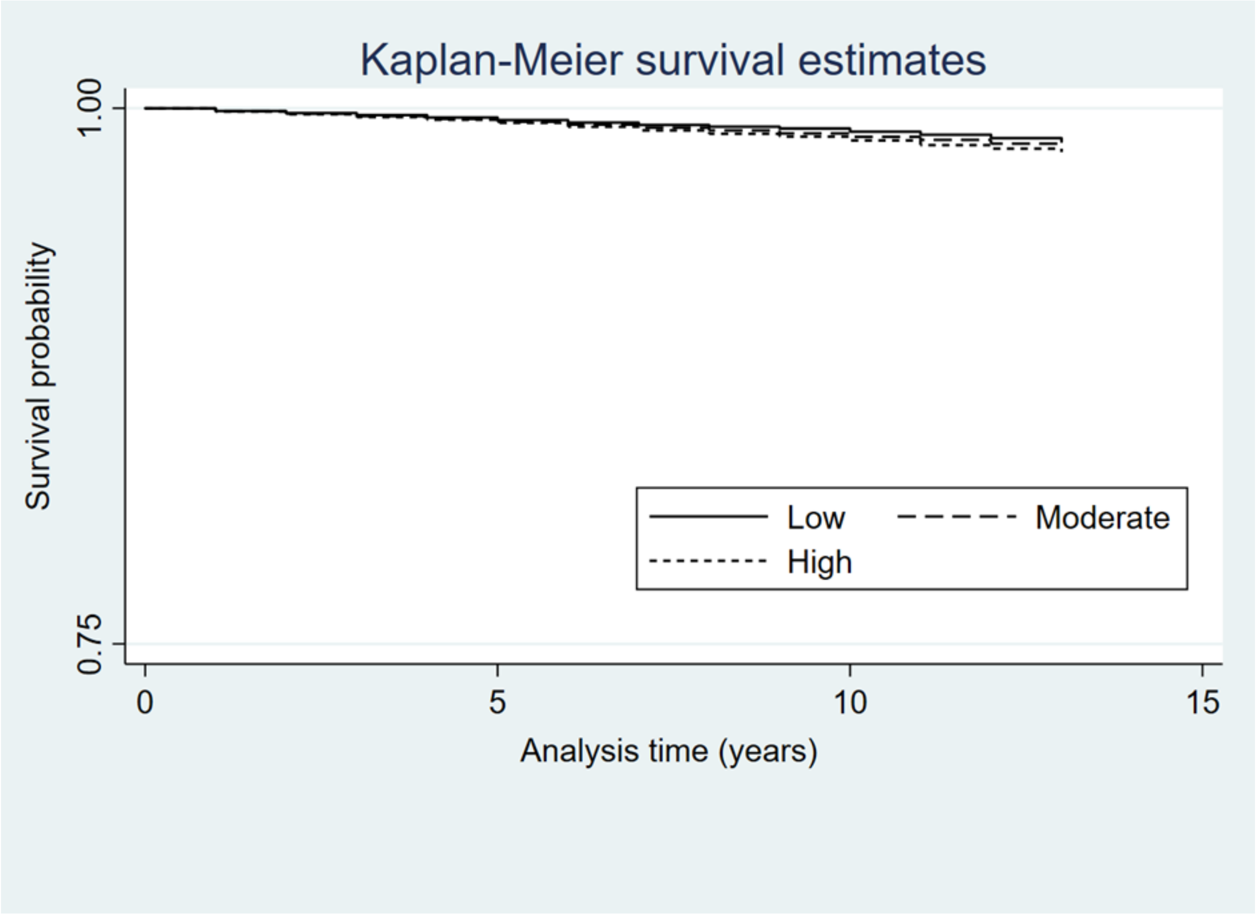

Supplement: S2 Fig — (PDF) [file pone.0288959.s002.pdf]
