# Supplementary material for: Neighborhood deprivation in relation to lung cancer in individuals with type 2 diabetes—A nationwide cohort study (2005–2018)
Source: PLoS One. 2023 Jul 21;18(7):e0288959. doi: 10.1371/journal.pone.0288959 (PMC10361504; doi:10.1371/journal.pone.0288959)

S3 Fig. Hazard ratios (HR) and 95% confidence intervals (CI) for incidence for lung cancer in men and women with type 2 diabetes

HR: Hazard ratio; CI: Confidence interval.

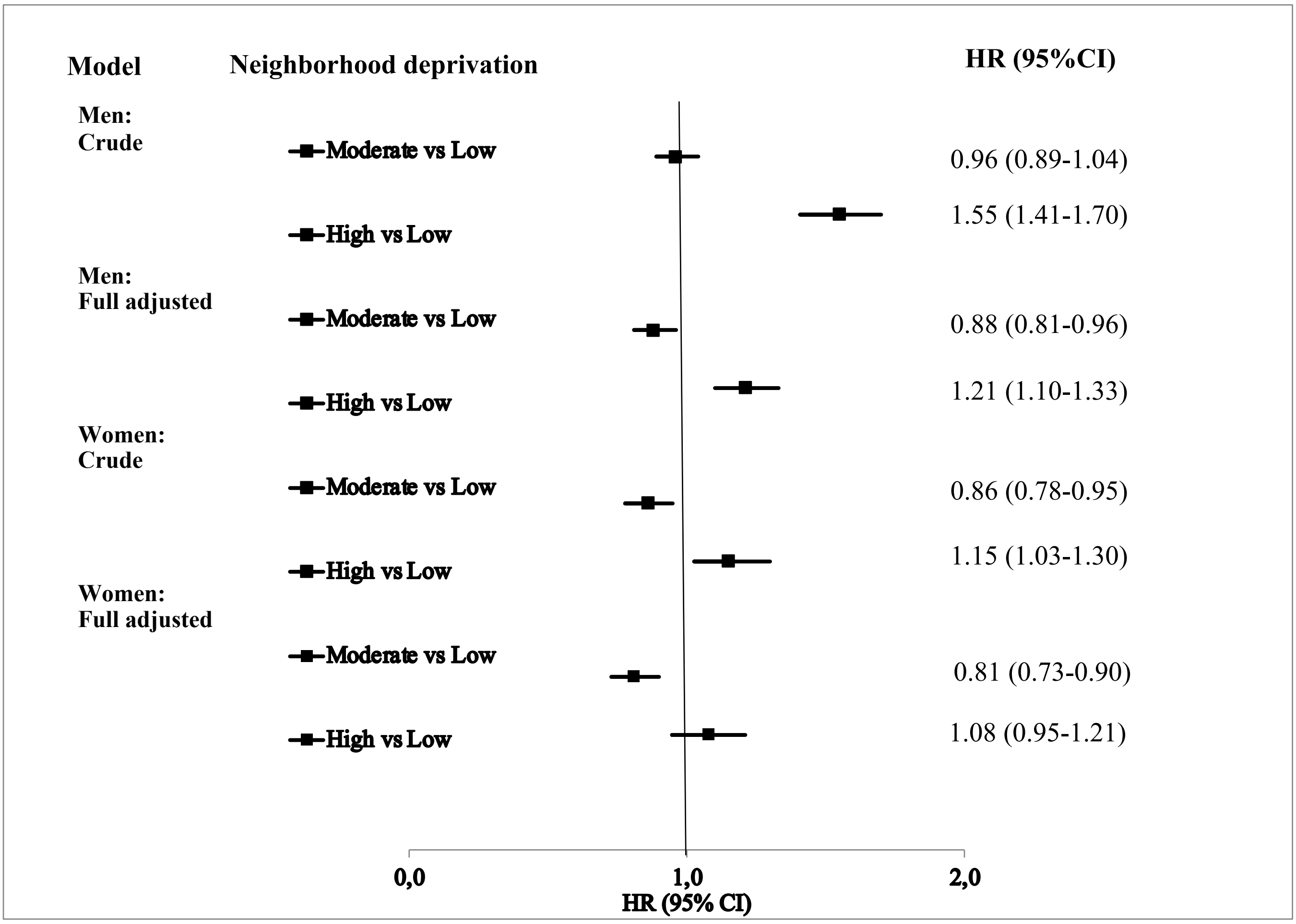

Supplement: S3 Fig — (PDF) [file pone.0288959.s003.pdf]
