# Supplementary material for: Neighborhood deprivation in relation to lung cancer in individuals with type 2 diabetes—A nationwide cohort study (2005–2018)
Source: PLoS One. 2023 Jul 21;18(7):e0288959. doi: 10.1371/journal.pone.0288959 (PMC10361504; doi:10.1371/journal.pone.0288959)
Supplement: S1 Table — (DOC) [file pone.0288959.s004.doc]

| **S1 Table.** ICD-codes of diagnoses and ATC-codes for treatments | | |
| --- | --- | --- |
| **Variables** | **Codes** | **Registers** |
| Incidence lung cancer | ICD-7: 162, 163. | National Cancer Register |
| Diagnosis of diabetes | ICD-10: E11. | National Patient Register |
| Mortality of lung cancer | ICD-10: C33, C34. | The Swedish Cause of Death Register |
| Treatment of diabetes | ATC: A10. | National Prescribed Drug Register |
| Diagnosis of COPD | ICD-10: J40–J47. | National Patient Register |
| Diagnosis of alcoholism and related liver disorders | ICD-10: F10, K70. | National Patient Register |
| Diagnosis of tobacco use | ICD-10: F17, T65.2, Z71.6, Z72.0. | National Patient Register |
| Metformin treatment | ATC: A10BA02. A10BD02. A10BD05. A10BD07. A10BD08. A10BD10. A10BD11. A10BD13. A10BD14. A10BD15. A10BD16. A10BD20. A10BD22. A10BD23. A10BD25. | National Prescribed Drug Register |
| Cardiovascular mortality (competing risk) | ICD-10: I00-I9 | The Swedish Cause of Death Register |
| ATC: the Anatomic Therapeutic Chemical codes. COPD: Chronic obstructive pulmonary disease. ICD: the International Classification of Diseases. | | |
