# Supplementary material for: Neighborhood deprivation in relation to lung cancer in individuals with type 2 diabetes—A nationwide cohort study (2005–2018)
Source: PLoS One. 2023 Jul 21;18(7):e0288959. doi: 10.1371/journal.pone.0288959 (PMC10361504; doi:10.1371/journal.pone.0288959)
Supplement: S2 Table — (DOC) [file pone.0288959.s005.doc]

| **S2 Table.** Study population characteristics, number of lung cancer events and mortality for lung cancer (2005-2018) | | | | | | | |  |
| --- | --- | --- | --- | --- | --- | --- | --- | --- |
|  | Study Population | |  | Events of lung cancer | |  | Mortality of lung cancer | |
|  | No. | % |  | No. | % |  | No. | % |
| Total population | 613,650 |  |  | 6654 | 1.1 |  | 5687 | 0.9 |
| **Sex** |  |  |  |  |  |  |  |  |
| Males | 344,026 | 56.1 |  | 4100 | 61.6 |  | 3454 | 60.7 |
| Females | 269,624 | 43.9 |  | 2554 | 38.4 |  | 2233 | 39.3 |
| **Age (years)** |  |  |  |  |  |  |  |  |
| 30-49 | 76,863 | 12.5 |  | 105 | 1.6 |  | 76 | 1.3 |
| 50-59 | 118,650 | 19.3 |  | 904 | 13.6 |  | 679 | 11.9 |
| 60-69 | 176,746 | 28.8 |  | 2707 | 40.7 |  | 2099 | 36.9 |
| 70-79 | 148,316 | 24.2 |  | 2321 | 34.9 |  | 2061 | 36.2 |
| ≥ 80 | 93,075 | 15.2 |  | 617 | 9.3 |  | 772 | 13.6 |
| **Education attainment** |  |  |  |  |  |  |  |  |
| ≤ 9 years | 248,709 | 40.5 |  | 3274 | 49.2 |  | 3025 | 53.2 |
| 10–12 years | 242,612 | 39.5 |  | 2519 | 37.9 |  | 2011 | 35.4 |
| > 12 years | 122,329 | 19.9 |  | 861 | 12.9 |  | 651 | 11.4 |
| **Family income** |  |  |  |  |  |  |  |  |
| Low income | 153,378 | 25.0 |  | 1815 | 27.3 |  | 1723 | 30.3 |
| Middle-low income | 153,332 | 25.0 |  | 1824 | 27.4 |  | 1669 | 29.3 |
| Middle-high income | 153,510 | 25.0 |  | 1781 | 26.8 |  | 1438 | 25.3 |
| High income | 153,430 | 25.0 |  | 1234 | 18.5 |  | 857 | 15.1 |
| **Region of residence** |  |  |  |  |  |  |  |  |
| Large cities | 279,482 | 45.5 |  | 3125 | 47.0 |  | 2509 | 44.1 |
| Southern Sweden | 204,549 | 33.3 |  | 2035 | 30.6 |  | 1748 | 30.7 |
| Northern Sweden | 129,619 | 21.1 |  | 1494 | 22.5 |  | 1430 | 25.1 |
| **Marital status** |  |  |  |  |  |  |  |  |
| Married/cohabiting | 328,346 | 53.5 |  | 3815 | 57.3 |  | 3212 | 56.5 |
| Not married | 285,304 | 46.5 |  | 2839 | 42.7 |  | 2475 | 43.5 |
| **Country of origin** |  |  |  |  |  |  |  |  |
| Born in Sweden | 481,288 | 78.4 |  | 5310 | 79.8 |  | 4585 | 80.6 |
| Born in other countries | 132,362 | 21.6 |  | 1344 | 20.2 |  | 1102 | 19.4 |
| **Mobility** |  |  |  |  |  |  |  |  |
| Not moved | 470,391 | 76.7 |  | 5018 | 75.4 |  | 4186 | 73.6 |
| Moved | 143,259 | 23.3 |  | 1636 | 24.6 |  | 1501 | 26.4 |
| **Hospitalization for COPD** |  |  |  |  |  |  |  |  |
| No | 556,842 | 90.7 |  | 4972 | 74.7 |  | 4340 | 76.3 |
| Yes | 56,808 | 9.3 |  | 1682 | 25.3 |  | 1347 | 23.7 |
| **Hospitalization for alcoholism and related liver disorders** |  |  |  |  |  |  |  |  |
| No | 590,033 | 96.2 |  | 6348 | 95.4 |  | 5424 | 95.4 |
| Yes | 23,617 | 3.8 |  | 306 | 4.6 |  | 263 | 4.6 |
| **Hospitalization for tobacco abuse** |  |  |  |  |  |  |  |  |
| No | 604,735 | 98.5 |  | 6337 | 95.2 |  | 5476 | 96.3 |
| Yes | 8915 | 1.5 |  | 317 | 4.8 |  | 211 | 3.7 |

COPD: Chronic obstructive pulmonary disease.
