# Supplementary material for: Neighborhood deprivation in relation to lung cancer in individuals with type 2 diabetes—A nationwide cohort study (2005–2018)
Source: PLoS One. 2023 Jul 21;18(7):e0288959. doi: 10.1371/journal.pone.0288959 (PMC10361504; doi:10.1371/journal.pone.0288959)
Supplement: S3 Table — a. Cumulative incidence (%) for lung cancer in patients with type 2 diabetes by levels of neighborhood deprivation (2005–2018). b. Cumulative mortality (%) for lung cancer in patients with type 2 diabetes by levels of neighborhood deprivation (2005–2018). (DOC) [file pone.0288959.s006.doc]

| **S3a Table.** Cumulative incidence (%) for lung cancer in patients with type 2 diabetes by levels of neighborhood deprivation (2005-2018) | | | | | | | | | | | | | | | | | | | |
| --- | --- | --- | --- | --- | --- | --- | --- | --- | --- | --- | --- | --- | --- | --- | --- | --- | --- | --- | --- |
|  | **Low** | | |  | | **Moderate** | | | | |  | | **High** | | | | | | |
| **Neighborhood deprivation**: | **Incidence** | **95% CI** | |  | | **Incidence** | | **95% CI** | | |  | | **Incidence** | | **95% CI** | | | |  |
| Total population | 1.03 | 0.97 | 1.09 | |  | | 1.05 | | 1.02 | 1.08 | |  | | 1.21 | | 1.16 | 1.26 |  | |
| **Sex** |  |  |  | |  | |  | |  |  | |  | |  | |  |  |  | |
| Males | 1.10 | 1.03 | 1.17 | |  | | 1.15 | | 1.11 | 1.19 | |  | | 1.38 | | 1.32 | 1.44 |  | |
| Females | 0.94 | 0.85 | 1.03 | |  | | 0.92 | | 0.87 | 0.97 | |  | | 1.02 | | 0.94 | 1.09 |  | |
| **Age (years)** |  |  |  | |  | |  | |  |  | |  | |  | |  |  |  | |
| 30-49 | 0.17 | 0.00 | 0.60 | |  | | 0.14 | | 0.00 | 0.41 | |  | | 0.12 | | 0.00 | 0.47 |  | |
| 50-59 | 0.60 | 0.43 | 0.77 | |  | | 0.73 | | 0.64 | 0.82 | |  | | 0.94 | | 0.82 | 1.05 |  | |
| 60-69 | 1.38 | 1.29 | 1.46 | |  | | 1.47 | | 1.42 | 1.52 | |  | | 1.84 | | 1.76 | 1.91 |  | |
| 70-79 | 1.48 | 1.39 | 1.58 | |  | | 1.47 | | 1.41 | 1.52 | |  | | 1.94 | | 1.85 | 2.02 |  | |
| ≥ 80 | 0.71 | 0.54 | 0.88 | |  | | 0.58 | | 0.47 | 0.69 | |  | | 0.84 | | 0.68 | 0.99 |  | |
| **Education attainment** |  |  |  | |  | |  | |  |  | |  | |  | |  |  |  | |
| ≤ 9 years | 1.56 | 1.48 | 1.64 | |  | | 1.19 | | 1.14 | 1.24 | |  | | 1.43 | | 1.37 | 1.49 |  | |
| 10–12 years | 0.84 | 0.74 | 0.94 | |  | | 1.06 | | 1.01 | 1.11 | |  | | 1.15 | | 1.07 | 1.23 |  | |
| > 12 years | 0.69 | 0.57 | 0.82 | |  | | 0.70 | | 0.60 | 0.79 | |  | | 0.73 | | 0.59 | 0.87 |  | |
| **Family income** |  |  |  | |  | |  | |  |  | |  | |  | |  |  |  | |
| Low income | 1.56 | 1.45 | 1.66 | |  | | 1.00 | | 0.93 | 1.07 | |  | | 1.27 | | 1.20 | 1.56 |  | |
| Middle-low income | 1.05 | 0.92 | 1.18 | |  | | 1.18 | | 1.12 | 1.24 | |  | | 1.29 | | 1.21 | 1.05 |  | |
| Middle-high income | 1.07 | 0.96 | 1.18 | |  | | 1.16 | | 1.10 | 1.22 | |  | | 1.24 | | 1.14 | 1.07 |  | |
| High income | 0.72 | 0.61 | 0.83 | |  | | 0.84 | | 0.77 | 0.91 | |  | | 0.84 | | 0.69 | 0.72 |  | |
| **Region of residence** |  |  |  | |  | |  | |  |  | |  | |  | |  |  |  | |
| Large cities | 0.99 | 0.91 | 1.07 | |  | | 1.14 | | 1.09 | 1.19 | |  | | 1.19 | | 1.12 | 1.25 |  | |
| Southern Sweden | 0.88 | 0.76 | 0.99 | |  | | 1.00 | | 0.95 | 1.06 | |  | | 1.07 | | 0.97 | 1.16 |  | |
| Northern Sweden | 1.33 | 1.23 | 1.44 | |  | | 0.96 | | 0.89 | 1.03 | |  | | 1.47 | | 1.38 | 1.56 |  | |
| **Marital status** |  |  |  | |  | |  | |  |  | |  | |  | |  |  |  | |
| Married/cohabiting | 1.11 | 1.04 | 1.18 | |  | | 1.13 | | 1.09 | 1.18 | |  | | 1.28 | | 1.22 | 1.35 |  | |
| Not married | 0.91 | 0.81 | 1.00 | |  | | 0.96 | | 0.92 | 1.01 | |  | | 1.13 | | 1.06 | 1.20 |  | |
| **Country of origin** |  |  |  | |  | |  | |  |  | |  | |  | |  |  |  | |
| Born in Sweden | 1.03 | 0.97 | 1.09 | |  | | 1.05 | | 1.02 | 1.09 | |  | | 1.39 | | 1.33 | 1.45 |  | |
| Born in other countries | 1.05 | 0.90 | 1.19 | |  | | 1.05 | | 0.96 | 1.13 | |  | | 0.98 | | 0.90 | 1.06 |  | |
| **Mobility** |  |  |  | |  | |  | |  |  | |  | |  | |  |  |  | |
| Not moved | 0.96 | 0.89 | 1.03 | |  | | 1.07 | | 1.03 | 1.11 | |  | | 1.16 | | 1.10 | 1.22 |  | |
| Moved | 1.26 | 1.16 | 1.36 | |  | | 0.97 | | 0.90 | 1.05 | |  | | 1.33 | | 1.25 | 1.41 |  | |
| **Hospitalization for COPD** |  |  |  | |  | |  | |  |  | |  | |  | |  |  |  | |
| No | 0.87 | 0.81 | 0.93 | |  | | 0.88 | | 0.84 | 0.92 | |  | | 0.94 | | 0.88 | 0.99 |  | |
| Yes | 2.73 | 2.62 | 2.85 | |  | | 2.71 | | 2.64 | 2.77 | |  | | 3.69 | | 3.60 | 3.77 |  | |
| **Hospitalization for alcoholism and related liver disorders** |  |  |  | |  | |  | |  |  | |  | |  | |  |  |  | |
| No | 1.03 | 0.97 | 1.08 | |  | | 1.04 | | 1.01 | 1.08 | |  | | 1.20 | | 1.15 | 1.24 |  | |
| Yes | 1.12 | 0.84 | 1.40 | |  | | 1.24 | | 1.09 | 1.40 | |  | | 1.52 | | 1.32 | 1.72 |  | |
| **Hospitalization for tobacco abuse** |  |  |  | |  | |  | |  |  | |  | |  | |  |  |  | |
| No | 1.00 | 0.95 | 1.06 | |  | | 1.01 | | 0.98 | 1.05 | |  | | 1.16 | | 1.12 | 1.21 |  | |
| Yes | 3.88 | 3.58 | 4.18 | |  | | 3.46 | | 3.31 | 3.61 | |  | | 3.61 | | 3.41 | 3.80 |  | |

COPD: Chronic obstructive pulmonary disease.

| **S3b Table.** Cumulative mortality (%) for lung cancer in patients with type 2 diabetes by levels of neighborhood deprivation (2005-2018) | | | | | | | | | | | |  |  |
| --- | --- | --- | --- | --- | --- | --- | --- | --- | --- | --- | --- | --- | --- |
|  | **Low** | | |  | **Moderate** | | |  | **High** | | | | |
| **Neighborhood deprivation**: | **Mortality** | **95% CI** | |  | **Mortality** | **95% CI** | |  | **Mortality** | **95% CI** | | | |
| Total population | 0.85 | 0.79 | 0.91 |  | 0.89 | 0.85 | 0.93 |  | 1.08 | 1.03 | 1.12 | | |
| **Sex** |  |  |  |  |  |  |  |  |  |  |  | | |
| Males | 0.89 | 0.81 | 0.97 |  | 0.96 | 0.92 | 1.01 |  | 1.21 | 1.15 | 1.28 | | |
| Females | 0.80 | 0.70 | 0.90 |  | 0.80 | 0.74 | 0.86 |  | 0.92 | 0.84 | 0.99 | | |
| **Age (years)** |  |  |  |  |  |  |  |  |  |  |  | | |
| 30-49 | 0.17 | 0.00 | 0.60 |  | 0.09 | 0.00 | 0.42 |  | 0.07 | 0.00 | 0.51 | | |
| 50-59 | 0.48 | 0.29 | 0.67 |  | 0.54 | 0.43 | 0.64 |  | 0.70 | 0.57 | 0.83 | | |
| 60-69 | 0.99 | 0.88 | 1.09 |  | 1.13 | 1.08 | 1.19 |  | 1.52 | 1.44 | 1.61 | | |
| 70-79 | 1.28 | 1.18 | 1.38 |  | 1.30 | 1.25 | 1.36 |  | 1.75 | 1.67 | 1.84 | | |
| ≥ 80 | 0.78 | 0.62 | 0.94 |  | 0.73 | 0.63 | 0.83 |  | 1.16 | 1.03 | 1.29 | | |
| **Education attainment** |  |  |  |  |  |  |  |  |  |  |  | | |
| ≤ 9 years | 1.42 | 1.34 | 1.51 |  | 1.10 | 1.05 | 1.15 |  | 1.34 | 1.28 | 1.41 | | |
| 10–12 years | 0.67 | 0.56 | 0.78 |  | 0.85 | 0.79 | 0.90 |  | 0.93 | 0.84 | 1.02 | | |
| > 12 years | 0.47 | 0.32 | 0.62 |  | 0.52 | 0.42 | 0.63 |  | 0.63 | 0.48 | 0.79 | | |
| **Family income** |  |  |  |  |  |  |  |  |  |  |  | | |
| Low income | 1.60 | 1.50 | 1.70 |  | 0.90 | 0.82 | 0.97 |  | 1.23 | 1.16 | 1.31 | | |
| Middle-low income | 0.88 | 0.74 | 1.02 |  | 1.09 | 1.03 | 1.16 |  | 1.19 | 1.10 | 1.28 | | |
| Middle-high income | 0.81 | 0.68 | 0.93 |  | 0.97 | 0.90 | 1.03 |  | 0.98 | 0.87 | 1.09 | | |
| High income | 0.47 | 0.34 | 0.60 |  | 0.60 | 0.51 | 0.68 |  | 0.60 | 0.42 | 0.77 | | |
| **Region of residence** |  |  |  |  |  |  |  |  |  |  |  | | |
| Large cities | 0.74 | 0.65 | 0.83 |  | 0.93 | 0.88 | 0.98 |  | 0.97 | 0.90 | 1.05 | | |
| Southern Sweden | 0.69 | 0.56 | 0.82 |  | 0.87 | 0.81 | 0.93 |  | 0.94 | 0.84 | 1.03 | | |
| Northern Sweden | 1.35 | 1.25 | 1.46 |  | 0.85 | 0.77 | 0.93 |  | 1.51 | 1.42 | 1.61 | | |
| **Marital status** |  |  |  |  |  |  |  |  |  |  |  | | |
| Married/cohabiting | 0.94 | 0.87 | 1.02 |  | 0.92 | 0.87 | 0.97 |  | 1.15 | 1.09 | 1.22 | | |
| Not married | 0.71 | 0.60 | 0.82 |  | 0.86 | 0.81 | 0.91 |  | 0.99 | 0.92 | 1.06 | | |
| **Country of origin** |  |  |  |  |  |  |  |  |  |  |  | | |
| Born in Sweden | 0.85 | 0.78 | 0.91 |  | 0.89 | 0.86 | 0.93 |  | 1.30 | 1.24 | 1.36 | | |
| Born in other countries | 0.89 | 0.73 | 1.05 |  | 0.87 | 0.78 | 0.96 |  | 0.79 | 0.70 | 0.87 | | |
| **Mobility** |  |  |  |  |  |  |  |  |  |  |  | | |
| Not moved | 0.72 | 0.64 | 0.79 |  | 0.92 | 0.88 | 0.96 |  | 0.97 | 0.91 | 1.04 | | |
| Moved | 1.29 | 1.19 | 1.39 |  | 0.78 | 0.70 | 0.87 |  | 1.30 | 1.22 | 1.38 | | |
| **Hospitalization for COPD** |  |  |  |  |  |  |  |  |  |  |  | | |
| No | 0.73 | 0.67 | 0.80 |  | 0.76 | 0.72 | 0.80 |  | 0.86 | 0.80 | 0.92 | | |
| Yes | 2.11 | 1.98 | 2.24 |  | 2.15 | 2.08 | 2.23 |  | 3.04 | 2.94 | 3.13 | | |
| **Hospitalization for alcoholism and related liver disorders** |  |  |  |  |  |  |  |  |  |  |  | | |
| No | 0.85 | 0.79 | 0.91 |  | 0.88 | 0.85 | 0.92 |  | 1.06 | 1.01 | 1.11 | | |
| Yes | 0.85 | 0.53 | 1.17 |  | 1.10 | 0.94 | 1.27 |  | 1.32 | 1.10 | 1.53 | | |
| **Hospitalization for tobacco abuse** |  |  |  |  |  |  |  |  |  |  |  | | |
| No | 0.84 | 0.77 | 0.90 |  | 0.87 | 0.83 | 0.91 |  | 1.05 | 1.00 | 1.10 | | |
| Yes | 2.71 | 2.35 | 3.06 |  | 2.25 | 2.07 | 2.44 |  | 2.44 | 2.20 | 2.68 | | |
| COPD: Chronic obstructive pulmonary disease |  |  |  |  |  |  |  |  |  |  |  | | |
