# Supplementary material for: Neighborhood deprivation in relation to lung cancer in individuals with type 2 diabetes—A nationwide cohort study (2005–2018)
Source: PLoS One. 2023 Jul 21;18(7):e0288959. doi: 10.1371/journal.pone.0288959 (PMC10361504; doi:10.1371/journal.pone.0288959)
Supplement: S4 Table — (DOC) [file pone.0288959.s007.doc]

| **S4 Table.** Hazard ratios (HR) and 95% confidence intervals (CI) for mortality of lung cancer in men with diabetes mellitus; Results of Cox regression models | | | | | | | | | | | | | | | | | | |  | | |  |
| --- | --- | --- | --- | --- | --- | --- | --- | --- | --- | --- | --- | --- | --- | --- | --- | --- | --- | --- | --- | --- | --- | --- |
|  | Model 1 | | | |  | | Model 2 | | | | |  | | | Model 3 | | | | | | | |
|  | HR | 95% CI | |  | | HR | | 95% CI | | |  | | | HR | | | 95% CI | | | |  | |
| **Neighborhood deprivation (ref. Low)** |  |  |  |  | |  | |  |  |  | | |  | | |  | |  | |  | | |
| Moderate | 1.01 | 0.98 | 1.04 |  | | 0.86 | | 0.84 | 0.89 |  | | | 0.86 | | | 0.84 | | 0.89 | |  | | |
| High | 1.28 | 1.24 | 1.32 |  | | 1.05 | | 1.01 | 1.08 |  | | | 1.04 | | | 1.00 | | 1.07 | |  | | |
| **Age (ref. 30-49 years)** |  |  |  |  | |  | |  |  |  | | |  | | |  | |  | |  | | |
| 50-59 | 2.46 | 2.25 | 2.68 |  | | 2.84 | | 2.61 | 3.10 |  | | | 2.82 | | | 2.58 | | 3.07 | |  | | |
| 60-69 | 5.49 | 5.06 | 5.96 |  | | 6.57 | | 6.05 | 7.13 |  | | | 6.51 | | | 6.00 | | 7.07 | |  | | |
| 70-79 | 14.94 | 13.78 | 16.18 |  | | 16.13 | | 14.87 | 17.50 |  | | | 16.18 | | | 14.91 | | 17.54 | |  | | |
| ≥ 80 | 46.63 | 43.02 | 50.54 |  | | 46.05 | | 42.44 | 49.96 |  | | | 46.96 | | | 43.27 | | 50.96 | |  | | |
| **Education attainment (ref.> 12 years)** |  |  |  |  | |  | |  |  |  | | |  | | |  | |  | |  | | |
| ≤ 9 years |  |  |  |  | | 1.46 | | 1.41 | 1.50 |  | | | 1.44 | | | 1.40 | | 1.49 | |  | | |
| 10–12 years |  |  |  |  | | 1.18 | | 1.14 | 1.22 |  | | | 1.17 | | | 1.13 | | 1.21 | |  | | |
| **Family income ( ref. Highest quartiles)** |  |  |  |  | |  | |  |  |  | | |  | | |  | |  | |  | | |
| Low income |  |  |  |  | | 2.25 | | 2.17 | 2.32 |  | | | 2.22 | | | 2.14 | | 2.30 | |  | | |
| Middle-low income |  |  |  |  | | 1.68 | | 1.62 | 1.73 |  | | | 1.65 | | | 1.60 | | 1.71 | |  | | |
| Middle-high income |  |  |  |  | | 1.39 | | 1.35 | 1.44 |  | | | 1.37 | | | 1.33 | | 1.42 | |  | | |
| **Region of residence (ref. Large cities)** |  |  |  |  | |  | |  |  |  | | |  | | |  | |  | |  | | |
| Southern Sweden |  |  |  |  | | 1.01 | | 0.99 | 1.04 |  | | | 1.02 | | | 1.00 | | 1.04 | |  | | |
| Northern Sweden |  |  |  |  | | 1.37 | | 1.34 | 1.41 |  | | | 1.39 | | | 1.35 | | 1.42 | |  | | |
| **Marital status (ref. Married/cohabiting)** |  |  |  |  | | 1.22 | | 1.20 | 1.25 |  | | | 1.20 | | | 1.18 | | 1.23 | |  | | |
| **Country of origin (ref. Sweden)** |  |  |  |  | | 0.87 | | 0.84 | 0.89 |  | | | 0.87 | | | 0.84 | | 0.90 | |  | | |
| **Mobility (ref. Not moved)** |  |  |  |  | | 1.58 | | 1.55 | 1.62 |  | | | 1.56 | | | 1.53 | | 1.60 | |  | | |
| **Comorbidities** |  |  |  |  | |  | |  |  |  | | |  | | |  | |  | |  | | |
| Hospitalization of COPD (ref. Non) |  |  |  |  | |  | |  |  |  | | | 1.37 | | | 1.33 | | 1.42 | |  | | |
| Hospitalization of alcoholism and related liver disorders (ref. Non) | |  |  |  | |  | |  |  |  | | | 1.57 | | | 1.50 | | 1.64 | |  | | |
| Hospitalization of tobacco abuse (ref. Non) |  |  |  |  | |  | |  |  |  | | | 1.03 | | | 0.94 | | 1.14 | |  | | |
| Model 1: Adjusted for age; Model 2: Adjusted for individual sociodemographic characteristics; Model 3: Full model (incl. comorbidities). HR: Hazard ratio; CI: Confidence interval; COPD: Chronic obstructive pulmonary disease. | | | | | | | | | | | | | | | | | | | | | | |
