# Supplementary material for: Neighborhood deprivation in relation to lung cancer in individuals with type 2 diabetes—A nationwide cohort study (2005–2018)
Source: PLoS One. 2023 Jul 21;18(7):e0288959. doi: 10.1371/journal.pone.0288959 (PMC10361504; doi:10.1371/journal.pone.0288959)
Supplement: S5 Table — (DOC) [file pone.0288959.s008.doc]

| **S5 Table.** Hazard ratios (HR) and 95% confidence intervals (CI) for mortality of lung cancer in women with diabetes mellitus; Results of Cox regression models | | | | | | | | | | | | | | | | | | |
| --- | --- | --- | --- | --- | --- | --- | --- | --- | --- | --- | --- | --- | --- | --- | --- | --- | --- | --- |
|  | Model 1 | | |  | Model 2 | | | | |  | | | Model 3 | | | | |  |
|  | HR | 95% CI | |  | HR | 95% CI | | |  | | | HR | | 95% CI | | |  | |
| **Neighborhood deprivation (ref. Low)** |  |  |  |  |  |  |  |  | | |  | | |  |  |  | | |
| Moderate | 0.89 | 0.86 | 0.91 |  | 0.80 | 0.78 | 0.83 |  | | | 0.80 | | | 0.78 | 0.83 |  | | |
| High | 1.06 | 1.02 | 1.09 |  | 0.97 | 0.94 | 1.01 |  | | | 0.97 | | | 0.94 | 1.00 |  | | |
| **Age (ref. 30-49 years)** |  |  |  |  |  |  |  |  | | |  | | |  |  |  | | |
| 50-59 | 3.51 | 3.03 | 4.07 |  | 3.92 | 3.38 | 4.54 |  | | | 3.88 | | | 3.35 | 4.50 |  | | |
| 60-69 | 8.00 | 6.96 | 9.20 |  | 8.70 | 7.57 | 10.01 |  | | | 8.69 | | | 7.56 | 10.00 |  | | |
| 70-79 | 23.81 | 20.75 | 27.31 |  | 23.33 | 20.33 | 26.78 |  | | | 23.65 | | | 20.61 | 27.15 |  | | |
| ≥ 80 | 90.17 | 78.64 | 103.38 |  | 80.95 | 70.54 | 92.90 |  | | | 83.46 | | | 72.72 | 95.79 |  | | |
| **Education attainment (ref.> 12 years)** |  |  |  |  |  |  |  |  | | |  | | |  |  |  | | |
| ≤ 9 years |  |  |  |  | 1.43 | 1.38 | 1.49 |  | | | 1.42 | | | 1.37 | 1.48 |  | | |
| 10–12 years |  |  |  |  | 1.09 | 1.05 | 1.14 |  | | | 1.08 | | | 1.04 | 1.13 |  | | |
| **Family income (ref. Highest quartiles)** |  |  |  |  |  |  |  |  | | |  | | |  |  |  | | |
| Low income |  |  |  |  | 1.67 | 1.59 | 1.76 |  | | | 1.68 | | | 1.59 | 1.76 |  | | |
| Middle-low income |  |  |  |  | 1.44 | 1.37 | 1.52 |  | | | 1.43 | | | 1.36 | 1.51 |  | | |
| Middle-high income |  |  |  |  | 1.17 | 1.11 | 1.24 |  | | | 1.16 | | | 1.10 | 1.23 |  | | |
| **Region of residence (ref. Large cities)** |  |  |  |  |  |  |  |  | | |  | | |  |  |  | | |
| Southern Sweden |  |  |  |  | 1.02 | 0.99 | 1.05 |  | | | 1.03 | | | 1.00 | 1.06 |  | | |
| Northern Sweden |  |  |  |  | 1.45 | 1.41 | 1.49 |  | | | 1.46 | | | 1.42 | 1.50 |  | | |
| **Marital status (ref. Married/cohabiting)** |  |  |  |  | 1.16 | 1.13 | 1.19 |  | | | 1.15 | | | 1.12 | 1.18 |  | | |
| **Country of origin (ref. Sweden)** |  |  |  |  | 0.86 | 0.83 | 0.89 |  | | | 0.86 | | | 0.83 | 0.89 |  | | |
| **Mobility (ref. Not moved)** |  |  |  |  | 1.71 | 1.67 | 1.75 |  | | | 1.71 | | | 1.66 | 1.75 |  | | |
| **Comorbidities** |  |  |  |  |  |  |  |  | | |  | | |  |  |  | | |
| Hospitalization of COPD (ref. Non) |  |  |  |  |  |  |  |  | | | 1.41 | | | 1.36 | 1.45 |  | | |
| Hospitalization of alcoholism and related liver disorders (ref. Non) |  |  |  |  |  |  |  |  | | | 1.76 | | | 1.60 | 1.93 |  | | |
| Hospitalization of tobacco abuse (ref. Non) |  |  |  |  |  |  |  |  | | | 1.19 | | | 1.05 | 1.34 |  | | |
| Model 1: Adjusted for age; Model 2: Adjusted for individual sociodemographic characteristics; Model 3: Full model (incl. comorbidities). HR: Hazard ratio; CI: Confidence interval; COPD: Chronic obstructive pulmonary disease. | | | | | | | | | | | | | | | | | | |
