# Supplementary material for: Neighborhood deprivation in relation to lung cancer in individuals with type 2 diabetes—A nationwide cohort study (2005–2018)
Source: PLoS One. 2023 Jul 21;18(7):e0288959. doi: 10.1371/journal.pone.0288959 (PMC10361504; doi:10.1371/journal.pone.0288959)
Supplement: S6 Table — (DOC) [file pone.0288959.s009.doc]

| **S6 Table.** Hazard ratios (HR) and 95% confidence intervals (CI) for incidence and mortality for lung cancer in patients diagnosed with type 2 diabetes with or without metformin treatment; Results of Cox regression models | | | | | | | | | | | | | |
| --- | --- | --- | --- | --- | --- | --- | --- | --- | --- | --- | --- | --- | --- |
|  | **With**  **metformin** | | | | | |  | | **Without metformin** | | | | |
|  | HR | | 95% CI | | | |  | | HR | | 95% CI | | |
| **Incidence of lung cancer** |  | |  | |  | |  | |  | |  | |  |
| Neighborhood deprivation (ref. Low) | |  | |  | |  | |  | |  | |  | |
| Moderate | 0.96 | | 0.88 | | 1.04 | |  | | 0.79 | | 0.71 | | 0.88 |
| High | 1.18 | | 1.07 | | 1.30 | |  | | 1.13 | | 1.01 | | 1.28 |
|  |  | |  | |  | |  | |  | |  | |  |
| **Mortality for lung cancer** |  | |  | |  | |  | |  | |  | |  |
| Neighborhood deprivation (ref. Low) | |  | |  | |  | |  | |  | |  | |
| Moderate | 1.01 | | 0.92 | | 1.12 | |  | | 0.70 | | 0.63 | | 0.79 |
| High | 1.27 | | 1.14 | | 1.42 | |  | | 1.16 | | 1.03 | | 1.31 |
| Fully adjusted for age, sex, individual sociodemographic characteristics, and comorbidities. | | | |  | |  | |  | |  | |  | |
