# Supplementary material for: Neighborhood deprivation in relation to lung cancer in individuals with type 2 diabetes—A nationwide cohort study (2005–2018)
Source: PLoS One. 2023 Jul 21;18(7):e0288959. doi: 10.1371/journal.pone.0288959 (PMC10361504; doi:10.1371/journal.pone.0288959)
Supplement: S7 Table — (DOC) [file pone.0288959.s010.doc]

| **S7 Table.** Hazard ratios (HR) and 95% confidence intervals (CI) for lung cancer incidence and mortality of individuals with type 2 diabetes with metformin treatment compared to individuals with type 2 diabetes without metformin treatment | | | | | | | |
| --- | --- | --- | --- | --- | --- | --- | --- |
|  | **Incidence lung cancer** | | |  | **Mortality lung cancer** | | |
|  | HR | 95% CI | |  | HR | 95% CI | |
| **All** | 0.65 | 0.61 | 0.68 |  | 0.56 | 0.53 | 0.60 |
| **Neighborhood deprivation** |  |  |  |  |  |  |  |
| Low | 0.61 | 0.54 | 0.69 |  | 0.52 | 0.45 | 0.59 |
| Moderate | 0.70 | 0.65 | 0.76 |  | 0.64 | 0.59 | 0.69 |
| High | 0.64 | 0.58 | 0.71 |  | 0.54 | 0.48 | 0.60 |

Fully adjusted for age, sex, individual sociodemographic characteristics, and comorbidities.
