# Supplementary material for: Neighborhood deprivation in relation to lung cancer in individuals with type 2 diabetes—A nationwide cohort study (2005–2018)
Source: PLoS One. 2023 Jul 21;18(7):e0288959. doi: 10.1371/journal.pone.0288959 (PMC10361504; doi:10.1371/journal.pone.0288959)
Supplement: S8 Table — (DOC) [file pone.0288959.s011.doc]

| **S8 Table.** Hazard ratios (HR) and 95% confidence intervals (CI) for incidence and mortality of lung cancer with competing risk for cardiovascular mortality; Results of Cox regression models | | | | | | | |  | |
| --- | --- | --- | --- | --- | --- | --- | --- | --- | --- |
|  | **Incidence lung cancer** | | |  | **Mortality lung cancer** | | |  | |
|  | HR | 95% CI | |  | HR | 95% CI | |  | |
| **Neighborhood deprivation (ref. Low) [men and women]** |  |  |  |  |  |  |  |  | |
| Moderate | 0.84 | 0.82 | 0.85 |  | 0.83 | 0.82 | 0.85 |  | |
| High | 1.00 | 0.98 | 1.03 |  | 1.00 | 0.98 | 1.03 |  | |
| **Neighborhood deprivation (ref. Low) [men]** |  |  |  |  |  |  |  |  | |
| Moderate | 0.87 | 0.85 | 0.89 |  | 0.86 | 0.84 | 0.89 |  | |
| High | 1.04 | 1.00 | 1.07 |  | 1.04 | 1.00 | 1.07 |  | |
| **Neighborhood deprivation (ref. Low) [women]** |  |  |  |  |  |  |  |  | |
| Moderate | 0.81 | 0.78 | 0.83 |  | 0.80 | 0.78 | 0.83 |  | |
| High | 0.97 | 0.93 | 1.00 |  | 0.97 | 0.94 | 1.00 |  | |
| HR: Hazard ratio; CI: Confidence interval. Fully adjusted for all covariates (age, individual-level sociodemographic factors and comorbidities). | | | | | | | | |  |
